# Corticospinal Facilitation during Observation of Graspable Objects: A Transcranial Magnetic Stimulation Study

**DOI:** 10.1371/journal.pone.0049025

**Published:** 2012-11-08

**Authors:** Michele Franca, Luca Turella, Rosario Canto, Nicola Brunelli, Luisa Allione, Nico Golfré Andreasi, Marianna Desantis, Daniele Marzoli, Luciano Fadiga

**Affiliations:** 1 Department of Biomedical Sciences and Advanced Therapies, Section of Human Physiology, University of Ferrara, Ferrara, Italy; 2 Istituto Italiano di Tecnologia, Robotics, Brain and Cognitive Sciences Department, Genova, Italy; University of Bologna, Italy

## Abstract

In 1979, Gibson first advanced the idea that the sight of graspable objects automatically activates in the observer the repertoire of actions necessary to interact with them, even in the absence of any intention to act (“affordance effect”). The neurophysiological substrate of this effect was later identified in a class of bimodal neurons, the so-called "canonical" neurons, located within monkey premotor cortex. In humans, even if different behavioral studies supported the existence of affordance effect, neurophysiological investigations exploring its neural substrates showed contradictory results. Here, by means of Transcranial Magnetic Stimulation (TMS), we explored the time-course of the “affordance effect” elicited by the observation of everyday-life graspable objects on motor cortex of resting observers. We recorded motor evoked potentials (MEP) from three intrinsic hand muscles (two "synergic" for grasping, OP and FDI and one "neutral", ADM). We found that objects’ vision determined an increased excitability at 120 milliseconds after their presentation. Moreover, this modulation was proved to be specific to the cortical representations of synergic muscles. From an evolutionary perspective, this timing perfectly fits with a fast recruitment of the motor system aimed at rapidly and accurately choosing the appropriate motor plans in a competitive environment filled with different opportunities.

## Introduction

In his ecological approach to vision [1], Gibson argued that, when we look at objects, we directly perceive not only their physical properties, but also the constellation of potential actions we can perform on and with them. He called this set of potential actions “affordances” and stated that they are generated without the need or the intention to act on the observed object; at the same time, they are highly constrained by the observer’s motor repertoire. This theory suggests that a sensory-motor system, able to transform the physical properties of an object into motor commands at first sight and with minimum effort, is a pre-requisite for successfully interacting within a competitive environment.

A very strong support to the affordance idea comes from neurophysiological studies in macaque monkeys, showing a cortico-cortical network devoted to transform object visual information into grasping actions which includes the anterior intraparietal region (AIP) and the ventral premotor cortex (in particular area F5). Premotor cortex, in turn, sends projections both to the primary motor cortex and to the cervical enlargement [2]–[6].

Rizzolatti and colleagues (1988), firstly reported a relevant population of neurons within premotor area F5, whose activity was strongly related to specific goal-directed actions such as grasping or manipulating specific objects [7]. The authors argued that motor neurons with different discharge properties code different goal-directed actions and all together they constitute a “motor vocabulary” always accessed by visual information. Among these motor neurons, about 20% showed object-related visual properties. More recently, area F5 visual properties were formally tested and it was described a set of bimodal visuomotor neurons with similar discharge pattern when a monkey grasps an object and when it simply watches a similar object without making any movement [8]. The visuomotor neurons belonging to this class have been successively named “canonical neurons” [9] to distinguish them from the other class of visuomotor neurons in area F5, the “mirror” neurons, responding instead to action observation [10]–[11]. Further studies confirmed the existence of “canonical” neurons within both ventral and dorsal premotor cortex [12]–[13], and within intraparietal region AIP and posterior parietal cortex [14]–[15].

Consistent with monkey evidence, behavioral studies in humans have demonstrated that the mere observation of a graspable object potentiates the observer's motor programs necessary to interact with it, even in absence of an explicit intention to act. This effect has been referred to as “visuomotor priming” or “affordance effect” [16]–[17]. In a series of experiment it was demonstrated that, when subjects were viewing an object with a handle oriented to the left or to the right, they reacted faster when the response hand was congruent with a given handle orientation, even if it was irrelevant for executing the task. The same authors further showed that Reaction Times (RTs) to visually presented large or small objects were significantly affected by the type of response executed by the participants (precision vs whole hand prehension), being faster in presence of congruence between response type and object affordance [18]–[20]. These results seem to confirm that, in the observer, the sight of the object recruits populations of neurons coding the motor program necessary to grasp it. The activity of such population may interact positively or negatively with the one coding the motor program of the selected hand response, both executed with the same muscular effectors.

Similar results have been successively confirmed and extended in the framework of the more general premotor theory of attention [17], [21]. Other authors described the time-course of the “affordance effect” adopting similar visual stimuli in the context of a Stimulus Onset Asynchrony (SOA) paradigm [22]. The reported effect was minimum for SOA = 0 and increased progressively with SOA, reaching its maximum for SOA = 800 and 1200. They thus concluded that the “affordance effect” develops gradually and persists for a relatively long period of time. These results were in agreement with a distributional analysis of reaction times performed in a prior study [19], allowing a further distinction from other Stimulus-Response Compatibility effects.

Human neuroimaging studies have consistently shown that, during the mere observation of graspable objects, a parieto-frontal circuit involved in visually guided grasping becomes significantly active. A Positron Emission Tomoraphy (PET) experiment with right-handed subjects, reported bilateral activation of premotor cortex during the observation of familiar tools [23]. A functional Magnetic Resonance Imging (fMRI) study, reported a correlation between the size of the “affordance effect” and the activity in the left posterior parietal and premotor cortices and showed increased activity in the left ventral precentral sulcus and in the left intraparietal cortex during the observation of object pictures [24]. Similar increased activations in ventral premotor and posterior parietal cortices (including the intraparietal sulcus and inferior parietal lobule) have been confirmed during the observation of pictures depicting tools [25]. Taken together, all these data seem to suggest that the regions within the dorsal stream automatically activated during object observation may constitute the neural substrate for the “affordance effect” originally hypothesized by Gibson.

Despite behavioral and brain imaging evidence, neurophysiological studies in humans have shown contradictory results. For example, Fadiga et al. [26] reported no effects on motor evoked potentials (MEPs) following single pulse transcranial magnetic stimulation (TMS) of motor cortex (M1), when objects were visually presented. However, TMS delivering was not finely time-locked with stimuli presentation and occurred with few seconds of delay with respect to the onset of object observation. More recently, a paired-pulse TMS protocol was applied to subjects preparing to grasp one of two possible objects, requiring different shaping of the hand and thus implying a different recruitment of the two recorded hand muscles, the first dorsal interosseus (FDI) and the abductor digiti minimi (ADM) [27]. The paired-pulse TMS protocol applied is known to produce higher MEPs, probably because it interacts with repetitive discharges of cortical output neurons [28]–[29] and, ultimately, it could be more effective than single-pulse TMS in revealing excitability differences at cortical level. In this experiment, TMS was delivered during motor preparation, well in advance of any visible electromyographic (EMG) activity, showing a facilitation pattern of MEPs which predicted the subsequent muscle activity during grasping for each object. The MEPs facilitation was absent during preparation to execute simple and complex intransitive movements and also during mere object presentation, not followed by a grasping action. This suggests that object vision does not induce any excitability change in the stimulated areas, unless an object-directed action is prepared. The same group replicated part of those results, failing in finding relevant differences in MEPs during object presentation alone [30]. Oppositely, in another TMS experiment [31], subjects were presented with pictures of familiar objects that could be normally grasped from a handle (tea-caps, tea-pots), oriented to the left or to the right and being thus compatible with a left-hand or right-hand grasp. It was also added a control condition comprising objects with broken handles and single TMS pulses were delivered to the left M1 200 milliseconds after stimuli onset. A significant contralateral MEPs facilitation was present only for objects with the handle oriented to the right side, therefore affording a movement of the right hand.

Recently, another group [32] used a combined approach to investigate the “affordance effect”: a reaction-time study and a TMS experiment, both comprising as stimuli, the presentation of pictures of familiar objects shown on a computer screen. They first replicated a behavioral study [19] by means of a similar apparatus to collect reaction times but, in addition to “pinchable” and “graspable” objects, they introduced a “neutral” condition with objects that could not be classified in any of the two previous groups (e.g sofa, carpet, door, etc). Behaviorally, a significant interaction between type of object and type of response was reported only for short SOA (400 milliseconds). In the TMS part of the experiment, they administered TMS at three different timings (300, 600, 900 milliseconds from stimulus onset) to the dominant M1 of subjects watching pictures selected from the behavioral experiment (“pinchable”, “graspable” and “neutral”) and recorded MEPs from FDI and ADM muscles, while participants were required to perform an attentional task not related to the presented object. The results showed a significant increase in MEPs amplitude congruent with the afforded grasp only for “pinchable” objects as compared only to “graspable” ones, if TMS was delivered 300 milliseconds after stimulus onset. No other significant differences were observed.

In the present paper, we decided to disentangle these ambiguous results by verifying if a significant modulation of M1 excitability dependent on objects observation is detectable in healthy human subjects by using TMS in a very controlled situation. To this aim, we used real objects as stimuli and required subjects to sit still and to relax while looking at them. So doing, no explicit movement preparation was affecting our results. Objects were always kept in front of each participant, within their peripersonal space. Objects were all small-sized, normally graspable with a grip involving the opposition of thumb and index finger (precision grip), while in the control condition everything was visually identical, apart from the presence of the object. TMS was applied to the left M1 of each subject and MEPs were recorded from three different intrinsic right hand muscles. Differently from previous studies, we decided here to investigate the time-course of corticospinal excitability in the time interval immediately following stimulus onset, at 120, 150 and 180 ms, prompted by the evidence that in monkey ventral premotor cortex, the majority of visuomotor neurons with object-related responses (“canonical” neurons) show a phasic peak of firing very soon after object presentation [8].

## Experiment 1

### Materials and Method

#### Subjects

Twenty-one healthy volunteers (8 males, 13 females; age 23–40) gave their written informed consent and participated in the study. The experimental procedures were approved by Ferrara University Ethics Committee and were performed according to the Declaration of Helsinki. All subjects had normal or corrected-to-normal vision and were naïve as to the aims of the experiment.

According to the Edinburgh Handedness Inventory [33], 19 participants were right-handed while 2 were left-handed. Prior to entering the study, the subjects were screened for possible adverse reaction to TMS [34] and were monetarily compensated at the end of the experiment.

#### EMG recordings and TMS

Surface EMG traces were recorded from three different intrinsic hand muscles of the right hand by using 9-mm-diameter adhesive Ag/AgCl surface electrodes (Kendall, GbmH, DE), in a belly-tendon montage. The muscles were the opponens pollicis (OP), the first dorsal interosseus (FDI) and the abductor digiti minimi (ADM), chosen because of their different involvement in grasping movements [27], [30]. Signals were amplified and band-pass filtered (50-1000 Hz) by means of a wireless EMG system (ZeroWire, Aurion s.r.l., IT), then digitized at 2000 Hz and stored on a PC for off-line analysis with Signal Software (2.02 Version, Cambridge Electronic Design, UK). EMG recordings started 200 milliseconds before visual stimuli presentation (which lasted 300 milliseconds) and ended 2 seconds after its conclusion (total duration 2.5 seconds).

Focal cortical stimulation was performed by means of a figure-of-eight coil (70 mm outer diameter) connected to a Magstim 200 magnetic stimulator (Magstim, Whitland, Dyfed, UK). Magnetic pulses had a nearly monophasic configuration, with a rise time of ∼100 µs, decaying back to zero over ∼0.8 milliseconds. The coil was positioned over the left motor cortex (M1), tangentially to the scalp, with the handle oriented 45° laterally and backwards with respect to the sagittal inter-hemispheric plane, inducing a medially and anteriorly directed current flow in the underlying cortex, approximately perpendicular to the central sulcus. First the hand motor area was localized and the “hot spot” for the right FDI defined as the scalp site, at which stimulation evoked the largest MEPs from the homonymous muscle. Then, the resting motor threshold for the FDI muscle (RMT) was identified, according to [35]. The stimulation intensity used in the experimental procedure was set to 120% of RMT of each subject to consistently evoke stable MEPs from all muscles in resting conditions.

#### Experimental procedure

**Figure 1:**
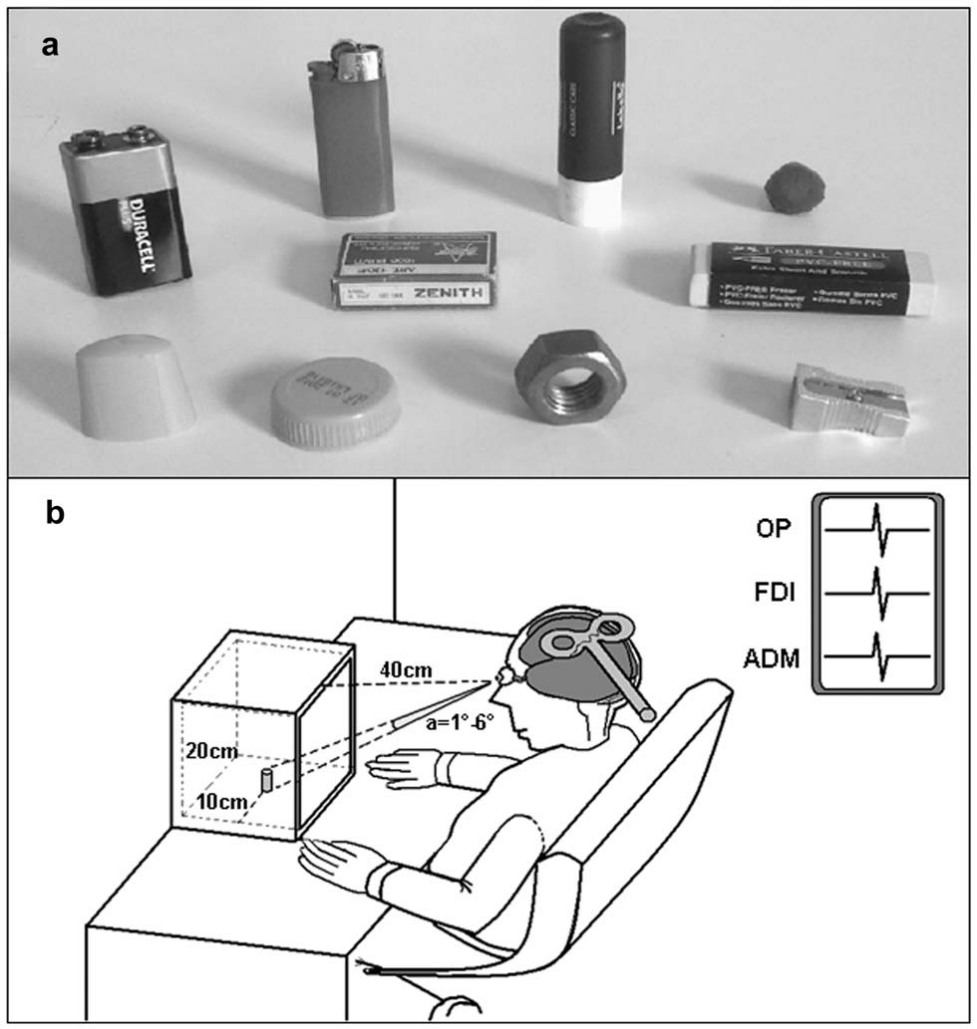
a. The set of objects presented. b. Schematic drawing depicting the adopted setup: 20-centimeter-sided square box (with an object inside) lying 40 cm in front of our subject. All the objects subtended a visual angle varying between 1° to 6° in width and height.

During the whole study, participants sat on a comfortable chair, with their hands resting pronated on a table in front of them. A 20×20×20 cm black box was placed on the table, its opening facing the subject at 40-centimeter distance and its upper edge being at the same height of the subject’s eyes. The centre of the floor of the box - where objects were placed in the “object” condition – coincided with subject’s mid-sagittal plane. There were two experimental condition: no-object (the box was left empty) and object (one object at a time was placed inside the box chosen from a pool of ten, see Fig. 1a). All objects were of similar, small size, to evoke similar type of grasping, i.e., thumb-to-index opposition (precision grip). Each object was presented 3 times for a total of 30 trials for object condition and 30 trials for the non-object one. We decided to test only small objects because of some evidence favoring the representation of precision grip over power grasp [36], [37]. With this setup (Fig. 1b), objects were within subject’s peripersonal space (i.e. reaching distance), and their 3D features (i.e. depth), were fully perceived even at the short viewing interval adopted. All objects were presented centrally and with the longest dimension facing the subjects, so that they were all subtended by a visual angle raging between 1° and 6° in height and width. The background luminance level of both stimuli (empty box and box with object inside) was equal to 0.3 Lux, as measured at the open face of the box with a photometer (Reed ST-1301 Light Meter).

**Figure 2:**
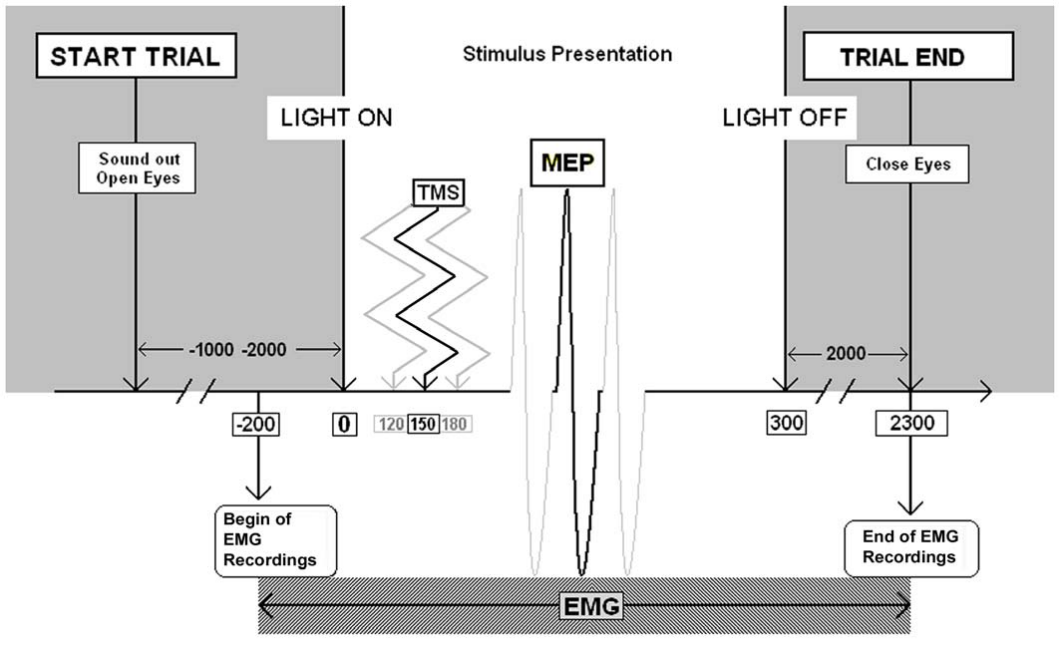
Schematic time-line of events in each trial. All numbers indicate milliseconds relative to the turning on of the LED inside the box (time 0). In black, magnetic pulse delivered 150 milliseconds after stimulus presentation onset, while alternative delays of magnetic stimulation are depicted in light grey.

Participants were required to keep their head on a chinrest, while TMS coil was kept in place by a mechanical arm (Manfrotto, IT). This guaranteed constant head and coil positions throughout the study. The room was completely darkened before the session started. Every trial was structured as follows (Fig. 2): a warning sound advised the subject to open his/her eyes. After an interval of 1 to 2 seconds, the box with its content was illuminated for 300 milliseconds (Stimulus Presentation). A LED lamp inside the box, but hidden to the subject, lit the box up. Then, after one of three possible delays from stimulus onset (120, 150 or 180 milliseconds), a TMS pulse was delivered to the subject’s left. At the end of the trial, the subject had to close his/her eyes waiting for the next warning sound, while the stimulus was manually changed, according to a precompiled randomized list. Inter-trial interval was always longer than 10 seconds, avoiding any possible unwanted interference of one magnetic pulse on the next. Participants were requested to just watch the stimulus while keeping their hands relaxed in order to then answer a question asked after the end of the stimulus presentation (end of trial). The questions were about the presence or the absence of an object, or about its salient features (mainly color, shape, name or normal use). Questions related to manipulation or interaction with objects were explicitly avoided. By this way, corticospinal excitability as revealed by MEPs area, could not be influenced by any explicit ongoing motor program. Ten practice trials preceded the experimental procedure recordings. Randomization of trials was performed by E-Prime 2.0 Software, which also triggered EMG acquisition, magnetic stimulator and visual stimulus presentation (i.e. turning on and off the LED) via the adoption of the Usb to Parallel FIFO (UPF), [38].

#### Data analysis

Trials showing EMG activity prior to TMS were discarded from further analysis (less than 5% of all trials, equally distributed between the two conditions). After calculation on a trial by trial basis of MEPs area from rectified EMG, MEPs area values were normalized (z-scores) separately for every subject and for each muscle to reduce inter-subject variability. Offline analysis of MEPs area was carried out with MATLAB (version 9.a, The Mathworks Inc., Natick, USA) and STATISTICA (www.statsoft.com) to perform the statistical analysis. For each dependent variable, data regarding each muscle were entered in a 2×3 within-subject ANOVA. Main effects of Object Presence (two levels, object and no-object), TMS Delay (three levels, 120, 150 and 180 ms) and the interaction between these 2 factors were computed. Subsequently, paired t-test comparisons were performed between conditions of interest.

### Results

None of the subjects ever made any mistake answering questions related to the presented stimuli.

**Figure 3:**
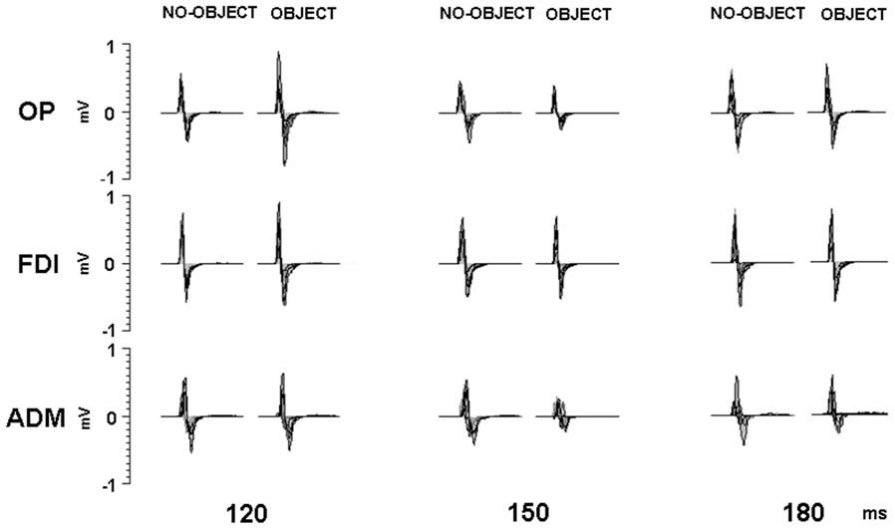
Effects of different conditions on MEPs, recorded in a representative subject. Each panel shows superimposed the traces (n = 10) evoked from the indicated intrinsic hand muscle.

Superimposed MEP traces from the three muscles of a representative subject are shown in Fig. 3 Mean MEP areas (+/−standard deviation) from OP, FDI and ADM across all subjects were equal to 10.9 (+/−11.8), 11.9 (+/−7.5), 7.1 (+/−4.8) mV*millisecond respectively.

#### Within-subject ANOVA for MEP area and post hoc comparisons

The within-subject ANOVA performed for the OP showed no significant result for the main effect of Object Presence (F_1,20_ = 2.520, p = 0.128) and for the main effect of TMS Delay (F_2,40_ = 1.011, p = 0.373), but the interaction was significant (F_2,40_ = 4.375, p = 0.019). Comparisons between the two conditions of interest (“object” and “no-object”) for each time interval revealed a significant difference for 120 ms delay (p<0.05).

For the FDI muscle, the main effect of Object Presence was significant (F_1,20_ = 5.086, p = 0.035), while TMS Delay and the interaction were not (F_2,40_ = 2.058, p = 0.141 and F_2,40_ = 0.469, p = 0.629, respectively). Post-hoc comparisons between object and no-object at the three delays revealed a significant difference for the 120 ms only (p<0.05).

**Figure 4:**
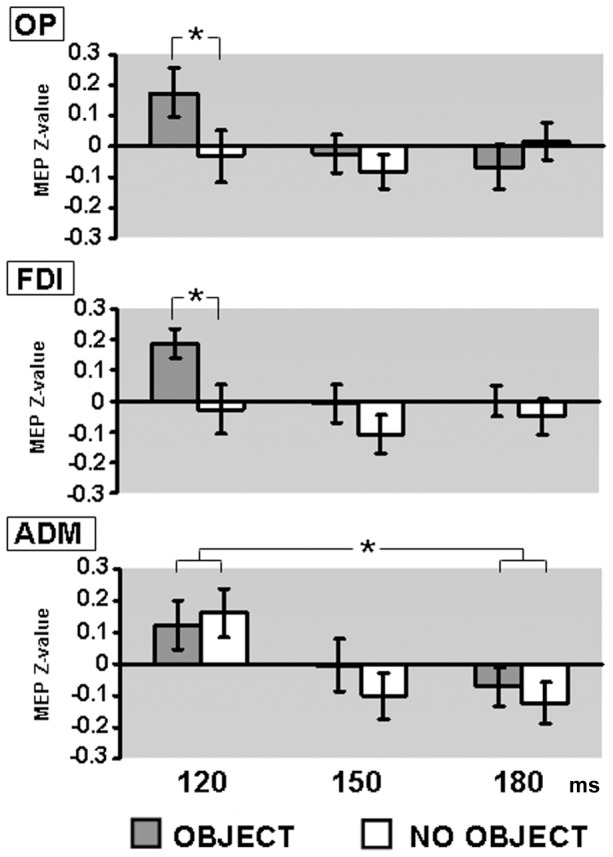
Graph representing normalized MEP data for three intrinsic hand muscles at the three timings studied (milliseconds from stimulus onset). From top to bottom of figure: opponens pollicis (OP), first dorsal interosseus (FDI), abductor digiti minimi (ADM). Dark grey histograms correspond to “object” condition, white histograms to “no-object” condition. Error bars indicate standard error of the mean. Asterisks indicate significant differences in post-hoc comparisons (p<0.05).

For the ADM muscle, while the main effect of Object Presence and the interaction were not significant (F_1,20_ = 0.177, p = 0.678 and F_2,40_ = 0.253, p = 0.778, respectively) the main effect of TMS Delay was significant (F_2,40_ = 3.719, p = 0.033). Post-hoc comparisons revealed a significant difference between 120 and 180 ms (p<0.05) independent from the presence of the object. Figure 4 summarizes the obtained results.

## Experiment 2

### Materials and Methods

#### Subjects

In another sample of ten subjects (8 female, 2 male; age 27–36), we verified the EMG activation of OP, FDI and ADM during the actual reaching and grasping of the objects adopted in the main TMS experiment. All volunteers were right-handed according to the Edinburgh Handedness Inventory [33], had normal or corrected to normal vision and were naïve as to the purposes of the study. An informed consent was obtained before recordings.

#### Experimental procedure

As in the TMS study, subjects sat with both hands resting pronated on a table in front or them and the same apparatus was adopted for stimuli presentation. Briefly, each one of the ten objects was placed one at a time in the center of the lower face of the black box, kept at 40 cm distance from participants. The room was dimly illuminated, and in each trial subjects were required to reach and grasp for the object at their own pace, to lift it for a few centimeters and to replace it in its starting position. All trials began with the turning on of the LED lamp that, illuminating the object, constituted the go-signal for participants. The trials ended 4 seconds later with the turning off of the light. Each object was presented five times consecutively (50 trials in total), and the surface EMG recordings of OP, FDI and ADM began 2 seconds before the go-signal, ending with the end of each trial. Intertrial interval was about 10 seconds. EMG traces were amplified, band-pass filtered (50-1000 Hz) and digitized at 2000 Hz and stored on a PC for off-line analysis. The order of object presentation was randomized across subjects. In order to analyze EMG activation until the object grasping phase, the first contact with the object was recorded through a touch-sensitive electrical circuit.

#### EMG data analysis

For each muscle, the rectified raw signal was integrated starting from the onset of significant EMG activity to 100 milliseconds after object contact (corresponding to the reaching and grasping phases). In this way, we avoided to include in the analysis subsequent lifting, holding and placing phases, necessary in order to record a purposeful grasping action. To better compare data from different subjects, in each trial we computed the ratio between the area during the contraction phase of each muscle, and the area in the equally lasting resting period that preceded contraction (turning on of light). Then, ratios from the three muscles were averaged across trials, and the averages from each subject were entered in a repeated measure ANOVA.

### Results

Mean ratio of EMG activation of OP, FDI and ADM across participants were respectively 18.69 (SE 2.81), 11.97 (SE 1.75) and 8.52 (SE 1.46). The repeated measure ANOVA reported a significant main effect of the factor Muscle (F_1,9_ = 3.4 p<0.001) and t-test post-hoc comparison showed that both OP and FDI were significantly more activated than ADM (p<0.05), while no significant differences could be reported between the two prime movers OP and FDI, during the pre-shaping and grasping phase of the objects presented, although a trend toward significance was clearly present (p = 0.075).

## Discussion

The main finding of the present study is that the motor system is automatically facilitated by the observation of graspable objects within a small time interval after their visual presentation. As proven by the second experiment, this facilitation was: muscle-specific, i.e. largely limited to the muscles mostly involved in the actual manipulation of the object (OP and FDI vs ADM); and it was time-specific, being present only in the earliest period of object observation (i.e. 120 milliseconds after its presentation). These results support the notion of a sensory-motor system automatically converting visual description of objects into pragmatic coordinates to act on them [2].

### Selectivity in Modulation of Corticospinal Excitability

Corticospinal excitability modifications reported in our study seem to reflect the sensory-motor activations that neuroimaging studies have already shown during object observation, along the parieto-frontal circuit for grasping [23]–[25], [39], [40]. Indeed, we were able to report a significant increase in MEP areas recorded from right FDI in the “object” respect to the “no- object” (control) condition, with a prominent modulation occurring in the first period of object observation, while noticeably, no differences could be found for ADM relatively to presented stimuli. Moreover, MEP recorded from OP revealed a significant interaction between stimuli and TMS-timing only 120 milliseconds after stimulus presentation. Finally, the last interesting result concerns the right ADM, whose MEP seem to reveal an increased excitability of its cortical representation limited to the first period of stimulus presentation, though independently from its nature (object vs no-object). The described excitability pattern across the representations of the three different muscles (OP, FDI, ADM) known to be widely intermingled in M1, reflects a transient cortical activation occurring simultaneously (i.e. 120 milliseconds from the instructing stimulus onset), but with a different level of sensitivity to the stimulus instructed movement. Indeed, 120 milliseconds after “object” stimulus onset, both right OP and FDI muscles excitabilities increased, while right ADM excitability did not show any stimulus specificity, being simply highest in the earliest period of stimuli presentation. These results rule out any unspecific attentional factor (e.g. arousal) as cause of the reported effect, avoiding as well the criticism, previously put forward, to explain the effect of object observation on motor cortices [41]. In this regard, it has to be noticed that differently from previous studies [17]–[20], [22], [31]–[32], the stimuli adopted here were all symmetrical, centrally presented and without differences in orientation. This means that although they possess fewer local spatial and functional differences with respect to objects with handles, they cannot evoke strong attentional spatial shifts, which in turn, would be expected to influence similarly OP, FDI and ADM.

A rather intuitive explanation to account for the present results would be that, as in monkeys [8], also in human subjects the sight of a graspable object recruits the same effectors that would be activated during the actual hand-object interaction, even if no action is planned. Indeed, during a precision grip on small-sized objects, a preferential increase in EMG activity would be expected in OP and FDI, with only a marginal increase in ADM [27], [51], paralleling the excitability modulations described, in the first 120 milliseconds of objects observation. The second experiment was conducted to verify this hypothesis. As expected, we could confirm that to successfully grasp the same objects shown in the first experiment, the EMG activity increased mostly in the OP and FDI, and only marginally in ADM. Taken together, results from both experiments demonstrate that excitability changes occurring in M1 of resting subjects, during the first 120 milliseconds of object observation, were congruent with the grip the objects would have “afforded” (i.e. cortical representations excitability increased due to object sight only in OP and FDI without differences in ADM).

We could have expected that the effect of object observation in M1, if already present after 120 milliseconds, were still present at later timings and maybe until the object were visible. This could be due to the fact that, as our task involved a simple detection of the object, the effect lasted until the detection of this stimulus was achieved. If the task had required a more complex cognitive process or the actual grasp of the object, the effect would have shown a longer persistence. Following this simple idea, it may be interesting to understand similarities and differences between the present findings and previously reported ones in terms of the adopted task and of the time-window in which the effect was present.

On the basis of the present study, it is tempting to say that a modulation dependent on the affordance is the most likely explanation. Nevertheless, one concern about what might seem a convincing conclusion could rise from the fact that the two class of stimuli adopted (empty box vs box with one of ten objects), beside the clear difference in “affordance” content (no-affordance vs precision grip), correlated with other differences such as low-level visual features (i.e. spatial frequency, color, contrast). So, we cannot rule out entirely the possibility that some difference between stimuli along one of those dimensions concurred in determining the results. However, it seems certainly less obvious what stimuli features, other than its “affordance”, might cause such peculiar pattern of excitability modulations at motor cortex as well as at peripheral muscular level. Furthermore, as visual features varied considerably with objects, they can hardly be expected to significantly influence the measured parameters (MEP and EMG activity).

### Comparison of the Present Study with Previous Investigations

Previous behavioral studies reported that the effect of affordance on reaction times increases slowly and lasts longer than what we observed in our data [22], [32], [42]. It should be noted, however, that the conclusions on activation timing that can be drawn from reaction time studies are not easily transferable to TMS experiments. In fact, in RTs studies, once the instruction-cue was recognized and the instructed response selected, the preparation-state was maintained until the go-signal. In this case, interactions between the motor representations related to the affordance and those coding the instructed motor response (at the basis of the effect) are more likely to occur for longer SOAs. Since in our study movements were to be suppressed, the time-window during which object-related modulation of M1 excitability occurred, vanished soon after object recognition, instead of lasting several hundreds of milliseconds after stimulus onset if a reaction is to be implemented. Furthermore, of relevance to the present discussion, an interference effect due to TMS pulses delivered on the dominant M1 was reported on EMG recordings from FDI and ADM on the subsequent self-paced reach-to-grasp movement only at 150 milliseconds from stimulus onset (TMS timings were 50, 100, 150 and 800 milliseconds after stimulus onset) [30]. Although different methodological issues suggest that a general conclusion could not be drawn from that result (as for example, about its validity in a reaction time paradigm), still they suggest that visual information processing about objects occurs in motor cortices from the very beginning of stimulus presentation. TMS interference lasted 50 milliseconds at most, and was already present for the 50 milliseconds-timing, vanishing apparently by the time of 150 milliseconds after stimulus onset (as proved by the absence of any interference in the two later timings). This could mean that the magnetic pulse prevented the stimulated area from receiving and processing object-related visual information necessary to select the following action, but only if delivered at the very beginning of object presentation, specifically before 150 milliseconds. As already mentioned, relevant differences in cortico-spinal excitability were absent at 50 and 100 milliseconds, but this does not surprise as they are probably too early for visual input to influence M1 excitability. TMS was shown to interfere with occipital cortex processing if delivered 100 milliseconds after visual stimuli onset [43], [44], and it seems conceivable to assume that if magnetic pulses on M1 interfere with its activity (in relation to object vision), they might do so only few milliseconds later. If a similar process had occurred in our subjects, then we should have expected a significant difference between “object” and “no-object” conditions also at 150 milliseconds and not only at 120 milliseconds. Nevertheless, as the subjects did not move, it is possible that M1 modulation due to object vision came up soon after the presentation started, lasting only for a very brief period, as intracortical inhibitory processes soon prevailed, dramatically shortening the time-window of the “affordance effect” reported by others. In this scenario, the sight of the object might have behaved as a “distractor”, pushing the excitability of M1 away from the resting/inhibited state required by the task, and closer to the action-related state. As a consequence, it is not too surprising to obtain an effect of affordance on MEPs around 120 milliseconds, dissipating soon afterwards. This implies a neural structure receiving object-related visual information and able to transform it into motor representations, in a very short time, and specifically by 120 milliseconds. In agreement with currently accepted models [45]–[46], this structure is likely to be represented by the parieto-frontal “dorsal” stream and within it, by the cortical areas with the most direct access to the primary motor cortex and to the spinal cord, specifically by premotor cortices. Single-cell recordings from monkey ventral premotor cortex have demonstrated the existence of neurons discharging during object presentation, as well as during the execution of the action needed to grasp a particular object [14]. Their discharge frequency pattern showed a phasic increase immediately after stimulus onset, peaking very soon. Furthermore, it was demonstrated that neurons from premotor cortex can influence the excitability of neurons in the ipsilateral primary motor cortex already after 1 millisecond (peaking in 6), through abundant and fast conducting cortico-cortical projections [47]–[48]. The same premotor-to-motor cortical circuit has been tested also in human subjects with the aid of bifocal TMS protocol, and the short latency of these influences was demonstrated on MEPs, and most interestingly excitability changes showed a state-dependent behavior [49]–[51]. Moreover, during naturalistic action observation (activating the “mirror neurons system”), it was recently described the close dependence on contextual information of afferent inputs that, originated in ventral premotor and posterior parietal cortex, synapse in M1 [52]–[53]. In this regard, it would be interesting to verify the time-course of interplay between different systems closely sited in the same cortical structures, such as the “canonical” and “mirror neuron system”, with a different specificity to visual stimuli (object vs action), focusing the attention to the earliest period of stimuli presentation, which proved to be the most relevant one in the present study. Although, we do not think that a system analogue to the “canonical neuron system” is the only explanation for the present results, we believe it could be one of the most probable candidates, because of its efferent connections to neurons in the primary motor cortex and because of its stimulus response properties (relevant stimulus features, phasic firing pattern, short latency increase in firing compatible with the early observed effect). Our results seem to be further supported by the recent observation of a significant activation of the sensorimotor cortex, contralateral to the forthcoming movement, 120 milliseconds after the instructing stimulus appearence [54]. Although different in many aspects, the methodologies implied (EEG and TMS) share similar time-resolution allowing a direct comparison [55]. The authors interpreted their results as dependent on the subject’s intention to act, as no activation could be detected during motor imagery tasks. The present work extend their results even further, strongly arguing against the occurrence of any effect due to the subject’s intention to act at the base of their and our results, as in our work no action had to be implemented. On the contrary, the differences observed in the present as well as in their work, can be explained more easily assuming that stimulus detection, in the motor system is “translated” into the motor program required by the task 120 milliseconds after its onset.

Finally, from an evolutionary perspective, an “affordance” effect at around 120 milliseconds seems to better fit the need of a rapid, accurate, on-line, visual information processing. As a consequence, this may be considered the most basic demonstration of the “affordance” effect, as it is evident in a simple detection task, whereas the effects reported in other studies may be caused by the same process (most likely canonical activation) persisting for the duration of more complex tasks, or by the combination of this process together with others more closely related to attention or motor preparation.

### Conclusions

In the present study, we reported evidence of an automatic recruitment of the dorsal stream during simple perception of graspable objects. This “affordance” effect was extremely selective both in terms of muscle activity and in terms of timing, suggesting that a “potential” action, specific for the observed object, was directly accessed following stimulus presentation.

It is tempting to interpret such effect within the theoretical framework of the “affordance competition hypothesis” recently proposed [46], where the processes of action selection and specification evolve simultaneously and continuously, and where different motor plans offered by the environment are progressively selected, through inhibitory processes, leading to the “winning” motor program to be implemented. The increased activity documented in motor areas by the present results due to graspable object observation, could be interpreted as the initial stage of preparation of motor programs needed to grasp the object, as a “potential” action to be executed. As the subjects know that no subsequent movement directed towards the object has to be initiated, the initial recruitment of the system may rapidly decay or may be actively inhibited in favor of other motor plans. Nevertheless, other possibilities cannot be excluded, as for example our evidence could describe the contribution of motor cortices to a more complete perception of graspable objects, especially when no action has to be implemented.
